# Synergistic Effect of Dual-Doped Carbon on Mo_2_C Nanocrystals Facilitates Alkaline Hydrogen Evolution

**DOI:** 10.1007/s40820-023-01135-0

**Published:** 2023-07-03

**Authors:** Min Zhou, Xiaoli Jiang, Weijie Kong, Hangfei Li, Fei Lu, Xin Zhou, Yagang Zhang

**Affiliations:** 1 School of Materials and Energy, University of Electronic Science and Technology of China, Chengdu, 611731 People’s Republic of China; 2 State Key Laboratory of Electronic Thin Films and Integrated Devices, University of Electronic Science and Technology of China, Chengdu, 610054 People’s Republic of China; 3 College of Physical Science and Technology, Yangzhou University, Yangzhou, 225002 People’s Republic of China; 4 Department of Chemical Engineering, Polytechnique Montréal, Montréal, QC H3C 3A7 Canada

**Keywords:** Molybdenum carbide, Hydrogen evolution reaction, Dual-doped, Synergistic effect, Superior performances

## Abstract

Molybdenum carbide (Mo_2_C) materials are promising electrocatalysts with potential applications in hydrogen evolution reaction (HER) due to low cost and Pt-like electronic structures. Nevertheless, their HER activity is usually hindered by the strong hydrogen binding energy. Moreover, the lack of water-cleaving sites makes it difficult for the catalysts to work in alkaline solutions. Here, we designed and synthesized a B and N dual-doped carbon layer that encapsulated on Mo_2_C nanocrystals (Mo_2_C@BNC) for accelerating HER under alkaline condition. The electronic interactions between the Mo_2_C nanocrystals and the multiple-doped carbon layer endow a near-zero H adsorption Gibbs free energy on the defective C atoms over the carbon shell. Meanwhile, the introduced B atoms afford optimal H_2_O adsorption sites for the water-cleaving step. Accordingly, the dual-doped Mo_2_C catalyst with synergistic effect of non-metal sites delivers superior HER performances of a low overpotential (99 mV@10 mA cm^−2^) and a small Tafel slope (58.1 mV dec^−1^) in 1 M KOH solution. Furthermore, it presents a remarkable activity that outperforming the commercial 10% Pt/C catalyst at large current density, demonstrating its applicability in industrial water splitting. This study provides a reasonable design strategy towards noble-metal-free HER catalysts with high activity.

**Supplementary Information:**

The online version contains supplementary material available at 10.1007/s40820-023-01135-0.

## Highlights

- The B and N dual-doped carbon layer that encapsulated on Mo_2_C nanocrystals (Mo_2_C@BNC) were fabricated for accelerating HER under alkaline condition.
- Theoretical calculations reveal that the H_2_O could be decomposed spontaneously over the introduced B sites, and the defective C atoms in the dual-doped carbon layer provide the best H binding sites.
- The optimized dual doped Mo_2_C catalyst with synergistic effect of non-metal sites delivers superior HER performances.

**Supplementary Information:**

The online version contains supplementary material available at 10.1007/s40820-023-01135-0.

## Graphical abstract

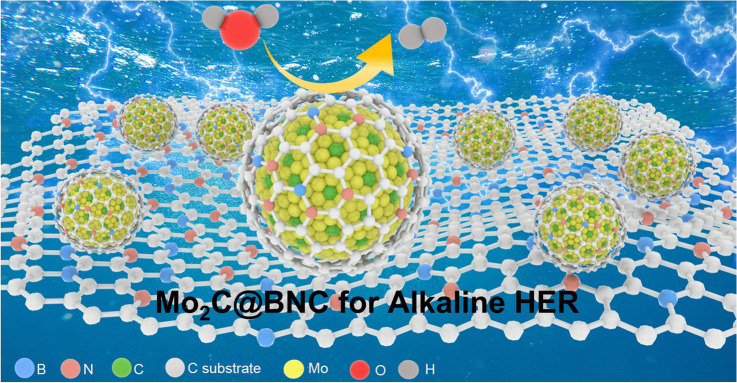

## Introduction

To address the threats of the energy crisis and environmental pollution from the increasing utilization of fossil fuels, great efforts have been devoted to exploring clean and renewable energy sources [1, 2]. Hydrogen, due to its high energy density, non-polluting and renewable features, has long been advocated as an excellent alternative to fossil fuels [3–5]. Electrocatalytic water splitting is considered as the most promising and desirable technology by researchers and entrepreneurs for hydrogen production [6–8]. So far, noble-metal-based materials (such as Pt, Ir and Ru-based catalysts) have been extensively investigated for hydrogen production. Unfortunately, the scarcity and high price limit their broad applications [9–14]. Therefore, one of the crucial tasks is the development of low-cost and high-performance noble-metal-free catalysts.

Molybdenum carbide (Mo_2_C) is emerging as an excellent replacement for the noble-metal-based catalysts by the virtue of low-cost and platinum-like electronic structure [15–18]. Recently, Yang et al. [19] proposed a novel coral-like porous nitrogen-doped Mo_2_C electrocatalyst (3D Mo_2_C) as HER electrocatalyst, which displayed an overpotential of 110 mV and a small Tafel slope of 73.9 mV dec^−1^ in 1 M KOH. Nevertheless, the strong hydrogen binding energy (HBE) of Mo_2_C hinders the further improvement of the HER activity, as the density of the *d*-orbital vacancies of Mo is relatively high [20, 21]. This favors the hydrogen adsorption process (Volmer step), but severely hampers the hydrogen desorption process (Heyrovsky or Tafel step) in HER processes.

Previous literatures have shown that coupling Mo_2_C with other transition metals (such as Co and Ni) is conducive to significantly enhance the HER performance via tuning the adsorption strength of the Mo–H bond [22, 23]. However, the metal dopants may suffer from dissolution issues in the electrolytes, causing ungratified stability. In this regard, carbon encapsulation onto the Mo_2_C can significantly improve the stability of the catalyst [24–26]. Meanwhile, a few studies shown that the exterior carbon can be activated by the interactions between Mo_2_C and the carbon encapsulated [27, 28]. More importantly, the alkaline HER kinetics is usually restricted by the water dissociation step, which may affect the whole reaction rate [29]. Therefore, the absence of water dissociation sites in Mo_2_C catalysts limited the further enhancement of the HER performance in alkaline medias. B-doping is previously proposed to benefit the water dissociation process. The electron-deficient B adsorbs water by coordinating with lone pair electrons, which weakens the O−H bond and thus accelerate the water dissociation step. For instance, Liu and Sun’s group demonstrated that B dopants in Ru NCs/BNG effectively boost the dissociation of water and facilitate the HER reaction rate [30].

Inspired by the previous results, this work reports the synthesis of B, N dual-doped carbon layer encapsulated on Mo_2_C nanocrystals (Mo_2_C@BNC) via a facile one-pot pyrolysis method for alkaline HER. The introduced B atom in the carbon layer is aimed to optimize the carbon shell electronic structures for H_2_O adsorption. As expected, the prepared Mo_2_C@BNC catalyst displays remarkable electrocatalytic activity towards HER under alkaline conditions. More importantly, the catalytic activity is even higher than that of the commercial 10% Pt/C catalyst at large current density. Theoretical calculations reveal that the H_2_O could be decomposed spontaneously over the introduced B sites, and the defective C atoms in the dual-doped carbon layer provide the best H binding sites. The synergistic effect between the non-metal sites (B and C) on the carbon shell optimize the rates of Volmer and Heyrovsky reaction, simultaneously, leading to the superb performance of the catalyst.

## Experimental Methods

### Experimental Reagents

Ammonium molybdate tetrahydrate ((NH_4_)_6_Mo_7_O_24_·4H_2_O), dicyandiamide (C_2_H_4_N_4_), boric acid (H_3_BO_3_), KOH are analytical grade and without further purification. Millipore water (resistivity: ∼ 18 MΩ cm) was used in all experiments.

### Synthesis of Mo_2_C@BNC

Mo_2_C@BNC was synthesized via two steps. First, 1 g of C_2_H_4_N_4_ was dissolved in 10 mL of deionized water, stirred for 15 min, then added 50 mg of (NH_4_)_6_Mo_7_O_24_·4H_2_O with 5 mg of H_3_BO_3_, heated and stirred until a white crystalline powder was formed. The white crystalline powder was collected and ground in an agate mortar for 15 min. In the second step, the powder was heated under an H_2_/Ar (5:95) atmosphere at 500 °C for 30 min with a heating rate of 5 °C min^−1^, then heated at 800 °C for another 6 h with a heating rate of 10 °C min^−1^.

### Synthesis of Mo_2_C@NC

The Mo_2_C@NC was prepared using the same method as the Mo_2_C@BNC except that the H_3_BO_3_ was not added.

### Synthesis of Mo_2_C@C

50 mg of (NH_4_)_6_Mo_7_O_24_·4H_2_O was mixed with 1 g of glucose and ground in an agate mortar for 15 min, then the homogeneous mixture was placed in a quartz tube and heated under an Ar atmosphere at 800 °C for 6 h with a heating rate of 5 °C min^−1^.

### Materials Characterizations

The phase structures of all samples were identified by Shimadzu X-ray diffraction (XRD)-7000 powder X-ray diffractometer (Cu Ka). The morphologies and structures of the products were characterized by Tecnai G2 F30 S-TWIN transmission electron microscopy (TEM). X-ray photoelectron spectroscopy (XPS) measurements were performed on a Thermo ESCALAB250Xi spectrometer with a resolution of 0.43 eV. Raman measurements were executed on a Renishaw inVia Raman spectrometer with a spectral range of 10 ~ 4000 cm^−1^. Fourier transform infrared spectroscopy (FT-IR) measurements were carried out on a Varian infrared spectrometer model Cary 670-IR with a wave number ranging from 4000 to 400 cm^−1^_._ The contact angle (CA) of the electrocatalysts on the electrode surface were measured using a contact angle meter (JY-82C).

### Electrochemical Measurements

The electrocatalytic activity was evaluated in a three-electrode system with 1 M KOH as electrolyte using a CHI 760E electrochemistry workstation (Chenhua, Shanghai). To obtain homogeneous ink, 1.2 mg acetylene black and 4.8 mg of sample were dispersed in 300 μL of ethanol and 30 μL of Nafion. The mixture was ultrasonicated for about 20 min, then added 300 μL of deionized water and sonicated for another 20 min. Next, 9 μL of the catalyst dispersion (8 mg mL^−1^) was loaded onto a glassy carbon electrode (0.1256 cm^2^ of area) and then dried at 60 °C for 10 min serving as the working electrode. In this case, the above prepared Mo_2_C@BNC was formulated as an ink drop on a L-shaped glassy carbon electrode (4 mm diameter) as the working electrode (the loading mass of Mo_2_C@BNC is 0.54 mg cm^−2^). Linear sweep voltammetry (LSV) was performed at a scan rate of 5 mV s^−1^. Electrochemical impedance spectroscopy (EIS) was performed at a cathode bias of − 0.2 V versus RHE, using a sinusoidal voltage of 5 mV in the range of 100 kHz–0.1 Hz. At 0.1–0.2 V versus RHE region, a series of CV measurements were performed at different sweep rates (10, 20, 40, 60, 80, 100 mV s^−1^). Stability was tested at 10 mA cm^−2^ for constant current V–t curves. The polarization curves normalized by ECSA was calculated on the basis of previous report with some modifications [31]. Firstly, *C*_dl_ determined from the slope of the plot between the charging current *i* and scan rate *v* (*C*_dl_ = *i*/*v*). Then, the ECSA calculated by dividing *C*_dl_ by specific capacitance (ECSA = *C*_dl_/*C*_s_). Here, the specific capacitances (*C*_s_) were chosen as *C*_s_ = 0.04 mF cm^−2^ based on typical reported values. Finally, the LSV curve was normalized by using the ECSA data obtained above.

### Density Functional Theory (DFT) Calculations

All DFT calculations were performed in the Vienna ab initio simulation package (VASP) using projector-augmented wave (PAW) potentials. The exchange–correlation energy is described by the generalized gradient approximation (GGA) with *Perdew-Burke-Ernzerholf* (PBE) functional. The cutoff value of the plane wave basis set is set to 400 eV. We employed a slab model where the vacuum spacing perpendicular to the surface layer is larger than 15 Å to eliminate physical interactions due to periodic boundary conditions along this direction. The convergence threshold of the force is reached when the Hellmann–Feynman force on each atom is less than 0.01 eV Å^−1^. 2 × 2 × 1 and 5 × 5 × 1 Γ-centered k-grids are used for geometric optimization and electronic structure calculations.

## Results and Discussion

### Catalyst Design and Structural Characterization

**Fig. 1:**
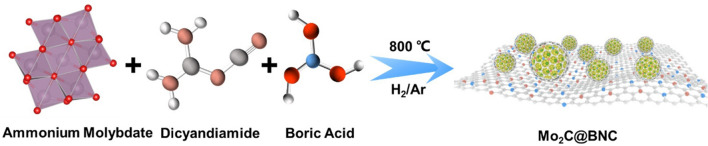
Schematic of synthesis N, B dual-doped Mo_2_C nanocrystals (Mo_2_C@BNC)

**Fig. 2:**
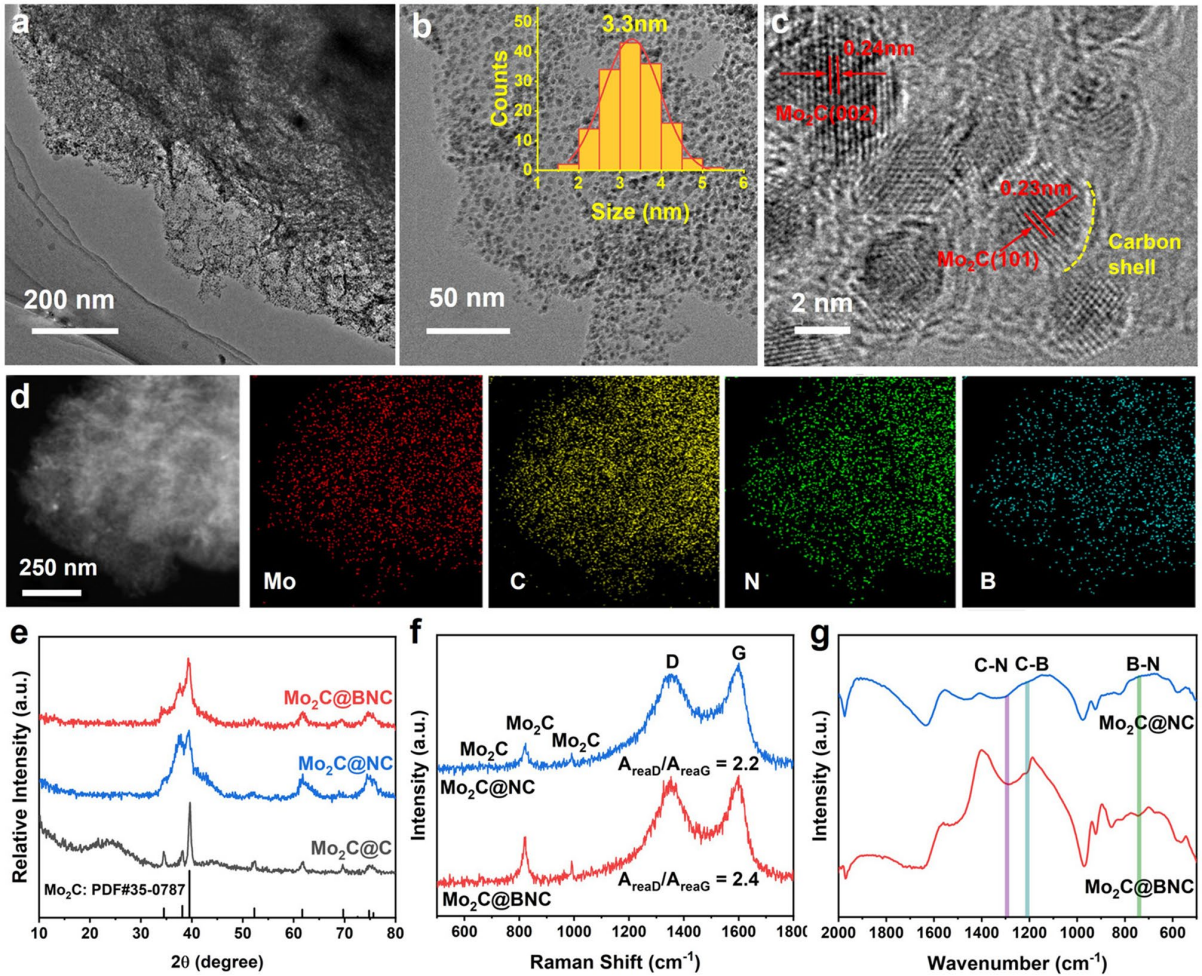
Structural characterization of the materials. **a**, **b** TEM images of the Mo_2_C@BNC, inset of **b**: the particle size distribution of the Mo_2_C nanocrystals. **c** HRTEM images of the Mo_2_C@BNC. **d** Elemental mapping of the Mo_2_C@BNC. **e** XRD pattern of the Mo_2_C@C, Mo_2_C@NC, and Mo_2_C@BNC. **f** Raman spectra of the Mo_2_C@NC and Mo_2_C@BNC. **g** FT-IR spectra of the Mo_2_C@NC and Mo_2_C@BNC

Mo_2_C@BNC was synthesized by a convenient one-pot pyrolysis method, in which ammonium molybdate tetrahydrate, dicyandiamide and boric acid were utilized as the raw materials (Fig. 1). The morphology of the sample was characterized by TEM and High-resolution TEM (HR-TEM). Figure 2a–b shows a foam-like structure of the Mo_2_C@BNC, in which Mo_2_C nanocrystals are uniformly distributed on the BNC substrate [32]. The inset of Fig. 2b displays the average particle size of ~ 3.3 nm for the Mo_2_C nanocrystals. The lattice spacing of 0.23 and 0.24 nm correspond to the (101) and (002) crystal planes of the Mo_2_C, respectively (Fig. 2c). Moreover, the Mo_2_C nanocrystals are found to be encapsulated by an ultrathin carbon shell (< 3 layers), which can effectively prevent the aggregation of Mo_2_C nanocrystals during the high-temperature pyrolysis process and protect the catalyst from corrosion by electrolyte during the catalytic reactions. It is noteworthy that, for a core–shell structure, the shell thickness is of significance to the catalytic activity of the catalysts. According to the reported literature [33], a thick shell (> 3 carbon layers) is not conducive to electron permeation to the surface of the catalyst. Hence, the ultrathin carbon shell in this work is beneficial for the electron transfer from the interior Mo_2_C to the carbon surface, which makes a charge redistribution on the exterior carbon shell. The bright spots in the high angle annular dark field scanning transition electron microscope (HAADF-STEM) image indicate the isolated Mo_2_C nanocrystals all over the BNC. The element mappings show that the Mo, C, N and B are homogeneously distributed (Fig. 2d). The Energy Dispersive X-Ray Spectroscopy (EDX) results show the doping contents of B and N are 10.32 and 8.42 wt% (Table S1), respectively, confirming the successful introducing of B and N elements into the catalyst. During the one-pot pyrolysis, the dicyandiamide not only serves as a nitrogen source to generate N-doped carbon shell that encapsulates the Mo_2_C nanocrystal, but also regulates the electronic structure of the catalyst surface and promotes the conductivity and stability of the catalyst. As control, when using glucose as the carbon source, the prepared Mo_2_C@C fails to form a regular morphology and exists in an amorphous material instead (Fig. S1).

XRD, FT-IR and Raman patterns were further employed to explore the detailed structure of the Mo_2_C@BNC. As shown in Fig. 2e, the peaks centered at 33.6°, 37.4°, 39.5°, 52.1°, 61.6° and 74.8° correspond to the (100), (002), (101), (102), (110) and (112) crystal planes of the hexagonal β-Mo_2_C (PDF#35-0787), respectively. No other peak can be observed in the Mo_2_C@BNC XRD pattern, indicating the boron and nitrogen mainly introduced into the carbon host of the Mo_2_C crystals. In the Raman spectra (Fig. 2f), vibration peaks around at 649.8, 818.7 and 991.1 cm^−1^ are fingerprint bands characteristic, confirming the formation of the Mo_2_C phase [34]. Additional peaks located at 1348 and 1595 cm^−1^ are attributed to the characteristic D and G bands of carbon materials, respectively. The *A*_reaD_/*A*_reaG_ ratio increases from 2.2 of the Mo_2_C@NC to 2.4 of the Mo_2_C@BNC, implying that the disorder of the graphite structure increases after the introduction of the extra B atoms [35]. The FT-IR spectrum of the Mo_2_C@BNC sample (Fig. 2g) shows a peak centered at 734 cm^−1^, corresponding to the out-of-plane vibration of the hexagonal B–N bond [36], which is not found in the Mo_2_C@NC spectrum. Besides, the peaks located at 1209.3 and 1297.6 cm^−1^ belong to the characteristic C−B bond and C−N bond, respectively [37, 38]. These results confirm the unique structure of the Mo_2_C@BNC, in which the Mo_2_C nanocrystals are encapsulated by an ultrathin B, N dual-doped carbon layer.

**Fig. 3:**
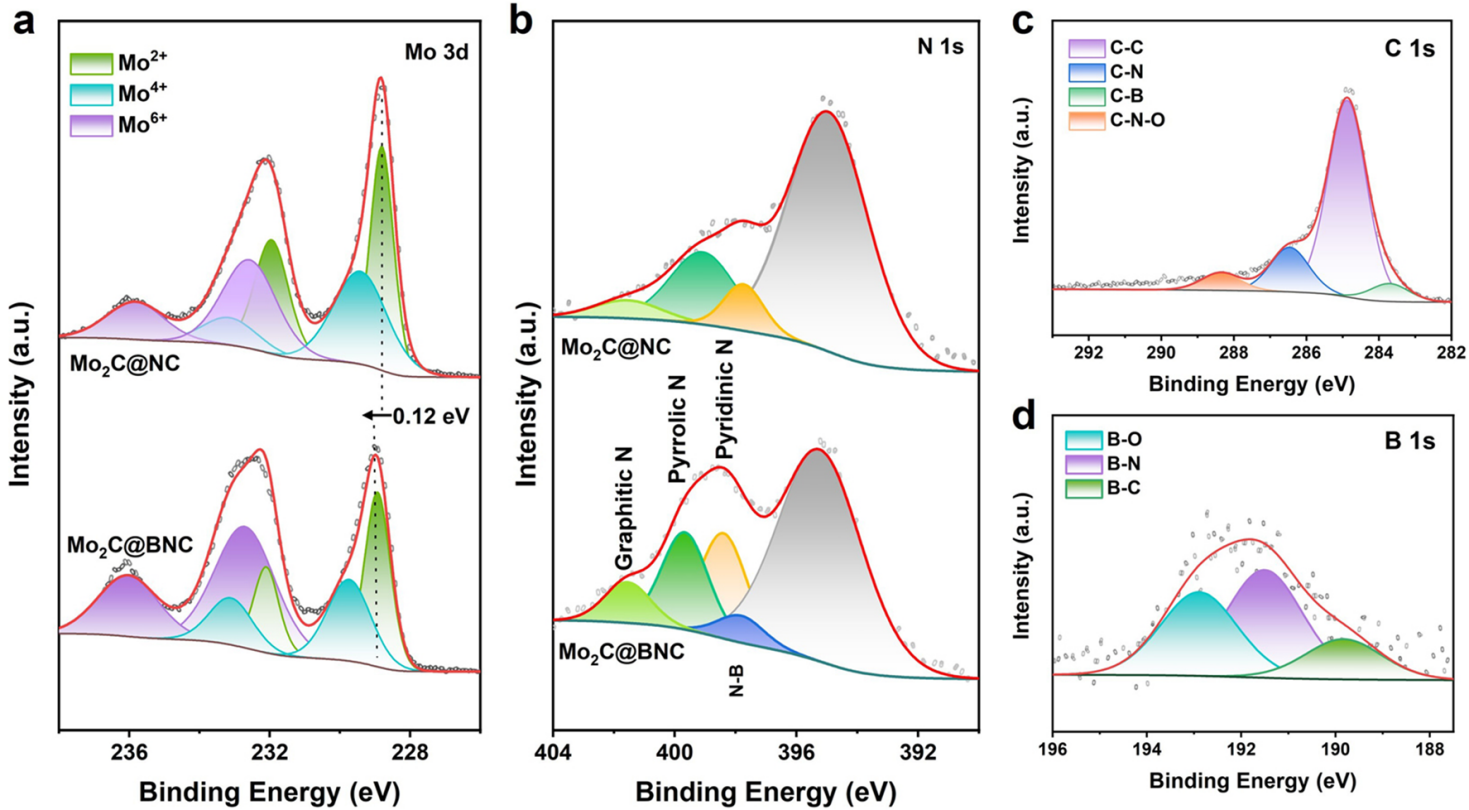
XPS spectra of Mo_2_C@NC and Mo_2_C@BNC: **a** Mo 3*d*. **b** N 1*s*. **c** C 1*s* XPS spectrum of Mo_2_C@BNC. **d** B 1*s* XPS spectrum of Mo_2_C@BNC

XPS analysis reveals the chemical states and electronic structures of the samples. In the Mo 3*d* spectra (Fig. 3a), the peaks at 228.9 and 231.98 eV correspond to the binding energies of Mo^2+^, which are typical characteristics of the Mo_2_C. The peaks at 229.89 and 233.26 eV correspond to the binding energies of Mo^4+^, while the peaks at 232.4 and 235.98 eV correspond to the binding energies of Mo^6+^, respectively, which are assigned to Mo species that oxidized in the preparation process [39, 40]. Compared with that of the Mo_2_C@NC sample, the Mo 3*d* XPS spectrum of the Mo_2_C@BNC presents a positive shift of 0.12 eV. It indicates the enhanced electron delocalization of the interior Mo_2_C after boron introduction, which facilitates the charge redistribution of the exterior BNC shell. In the N 1*s* spectrum (Fig. 3b), four peaks at 395.2, 398.4, 399.6 and 401.5 eV are observed, which can be assigned to the binding energies of Mo 3*p*, pyridinic N, pyrrolic N and graphitic N, respectively, further demonstrating the successful doping of N into the carbon layer. An additional peak is observed at 397.8 eV in the N 1*s* spectrum after B doping, which is attributed to the B-N binding energy [41]. In this work, according to the XPS spectrum, the pyridinic N content of the Mo_2_C@BNC is 38.97%, far greater than that of the Mo_2_C@NC (24.50%), implying that the electron structure of Mo_2_C@BNC has been greatly modified (Table S2). This means that the doping of B decorated the electronic structure of the exterior carbon shell. In the C 1*s* spectrum (Figs. 3c and S2), strong peaks can be observed for the C-C species at 284.8 eV, other peaks at 283.7, 286.4 and 288.3 eV correspond to the C-B, C-N and C-N-O binding energies [42]. The B 1*s* spectrum (Fig. 3d) can be observed with three peaks located at 189.8, 191.5 and 192.8 eV, corresponding to the B-C, B-N and B-O binding energies, respectively [43]. The co-existence of the B-C and B-N species indicates the partial replacement of C atoms with B atoms in the carbon layer. It’s also affirmed that partial electrons in the Mo_2_C@BNC are transferred from Mo to the carbon layer after the introduction of element B, and the dual-doped B and N elements enables the activation of the adjacent C atom due to electron restructuring, facilitating the hydrogen evolution reaction [44].

### Electrocatalytic Performance

**Fig. 4:**
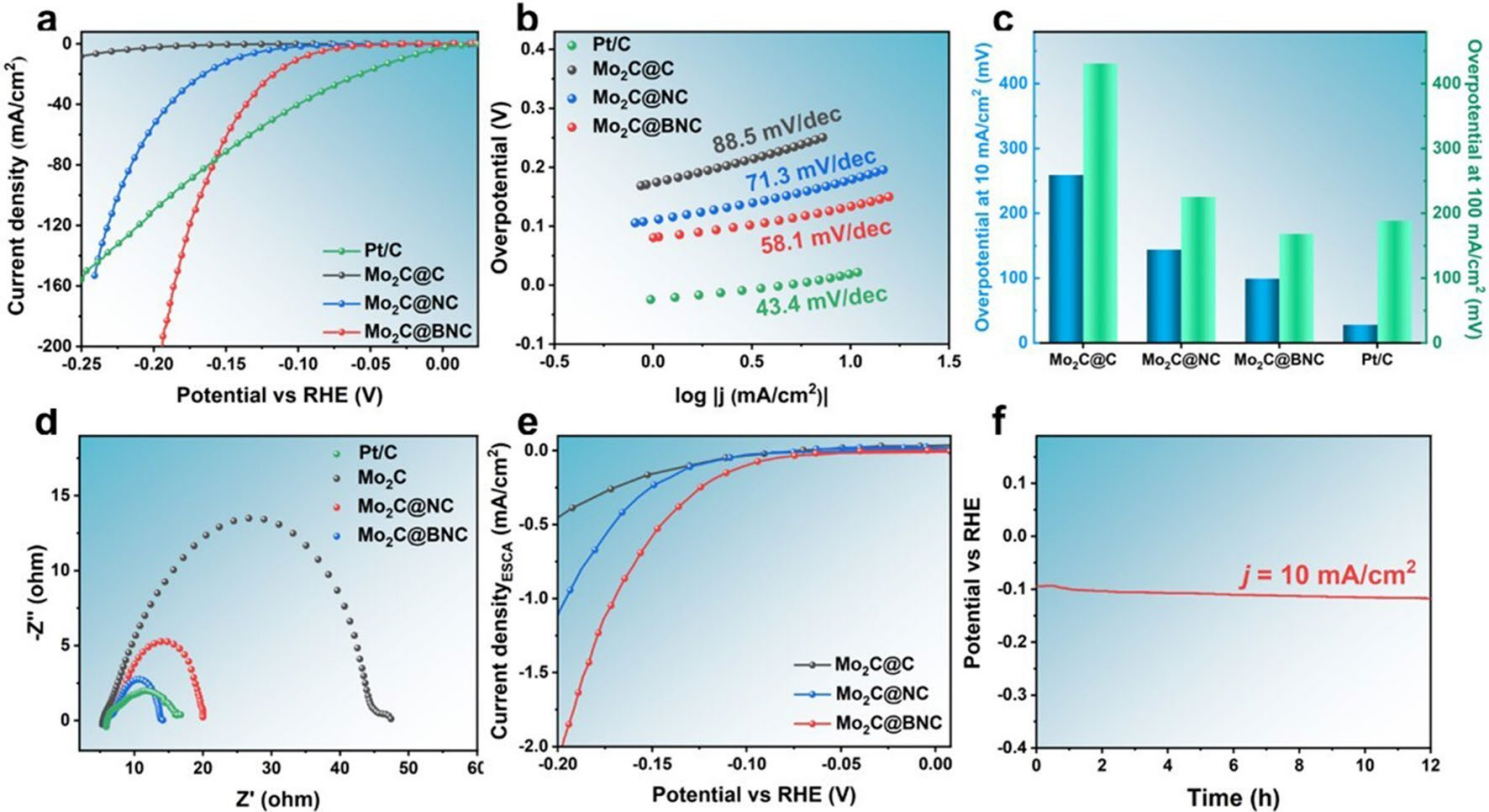
HER electrocatalytic properties of the Mo_2_C@BNC catalyst. **a** LSV curves of Mo_2_C@C, Mo_2_C@NC, Mo_2_C@BNC and Pt/C in 1 M KOH electrolyte. **b** Tafel slopes. **c** Overpotentials at 10 and 100 mA cm^−2^. **d** EIS curves. **e** The polarization curves normalized by ECSA. **f** Constant potential V–t curves of Mo_2_C@BNC

The HER activities of the prepared Mo_2_C@BNC catalyst were determined in 1 M KOH. The catalytic performances of the Mo_2_C@NC, Mo_2_C@C and commercial 10% Pt/C catalysts were also measured in the same condition as control. As shown in Fig. 4a, the designed Mo_2_C@BNC catalyst displays an excellent HER catalytic activity with an over potential of 99 mV to deliver the current density of 10 mA cm^−2^, which is significantly lower than those of the Mo_2_C@NC (144 mV) and Mo_2_C@C (259 mV), indicating that B doping can substantially enhance the HER electrocatalytic activity. We have also investigated the catalytic activity of the Mo_2_C@BNC catalysts with various B doping concentrations, provided an optimized B doping level in this study (Fig. S3). Moreover, the Mo_2_C@BNC shows a robust catalytic activity at large current density that even superior to the commercial 10% Pt/C catalyst (Fig. 4a, c). The overpotential is 168 mV for Mo_2_C@BNC to achieve 100 mA cm^−2^, 20 mV less than that of the commercial 10% Pt/C (188 mV), demonstrating its great potential in industrial applications. The performance of the Mo_2_C@BNC surpasses most state-of-the-art reported Mo_2_C-based materials (Table S3), addressing one of the best catalytic activities among Mo_2_C-based catalysts ever reported.

Furthermore, the Tafel slope is used to study the catalytic kinetics during the reaction process [45]. HER usually comprises two possible steps in alkaline media, as shown in the following equation:

$${\text{H}}_{2} {\text{O}} + {\text{e}}^- \to {\text{H}}^* + {\text{OH}}^- \;\;\;\;\left( {\text{Volmer reaction}} \right)$$

$${\text{H}}^* + {\text{H}}_{2} {\text{O}} + {\text{e}}^- \to {\text{H}}_{2} + {\text{OH}}^- \quad ({\text{Heyrovsky reaction}})$$

or

$${\text{H}}^* + {\text{ H}}^* \to {\text{H}}_{2} \quad ({\text{Tafel reaction}})$$

where H^*^ refers to the H atom adsorbed on the surface of the catalysts. HER is separated into the water-cleaving (Volmer reaction) and the hydrogen coupling (Tafel reaction or Heyrovsky reaction) steps, resulting in two reaction pathways, namely the Volmer–Heyrovsky or Volmer–Tafel mechanism. In general, the rate-determining step (RDS) can be understood by the Tafel slope, which reflects the electron transfer capacity between the H^*^ and the catalyst. The Tafel slope of 30 mV dec^−1^ shows that the RDS is Tafel step and the reaction follows the Volmer–Tafel pathway. The Tafel slope of 40 mV dec^−1^ indicates that the Heyrovsky step is the RDS and the reaction follows the Volmer–Heyrovsky pathway. The Tafel slope of 118 mV dec^−1^ means that Volmer step is the RDS and the reaction may follow the Volmer–Heyrovsky or Volmer–Tafel pathway [46]. As shown in Fig. 4b, the calculated Tafel slope of the Mo_2_C@BNC for HER is 58.1 mV dec^−1^, smaller than those of the Mo_2_C@NC (71.3 mV dec^−1^) and Mo_2_C@C (88.5 mV dec^−1^), demonstrating the superior kinetics of the Mo_2_C@BNC among the catalysts. The Tafel slope value of the Mo_2_C@BNC also implies that the reaction proceeds through a Volmer–Heyrovsky pathway, during which the Heyrovsky step is the rate-determining step. Meanwhile, we further confirm the superior adsorption of Mo_2_C@BNC on H by testing HER performance in acidic solution (Fig. S4). Besides, the EIS results (Fig. 4d) measured at − 0.2 *V*_RHE_ confirm that the Mo_2_C@BNC has the smallest charge-transfer resistance in the reaction.

The electrochemically active surface area (ECSA) of the catalyst is measured to further evaluate the effect of surface area on the electrochemical performance. ECSA is proportional to the electrochemical double-layer capacitance (*C*_dl_) of the material (Fig. S5). The Mo_2_C@BNC shows a great ECSA value, indicating that the B doping can expose more active sites and contribute to the HER catalytic activity [43]. Besides, the polarization curves were normalized by ECSA (J_ECSA_) to explore the effect of intrinsic activity, as shown in Fig. 4e. Mo_2_C@BNC exhibits the highest J_ECSA_ among all the samples, demonstrating the intrinsically enhanced activity. The stability of the Mo_2_C@BNC was tested by chronopotentiometry at a constant density of 10 mA cm^−2^ (Fig. 4f). There is a tiny increment of potential during the 12 h reaction, and no significant change in the morphology and structure of the catalyst after the durability test (Fig. S6), demonstrating the favorable stability of the catalyst. This is attributed to the fact that the carbon layer is wrapped with Mo_2_C nanocrystals, which can effectively prevent the electrolyte from corroding the catalyst and greatly maintain the catalytic activity [47].

### Mechanism Analysis of the Enhanced Catalytic Performance

**Fig. 5:**
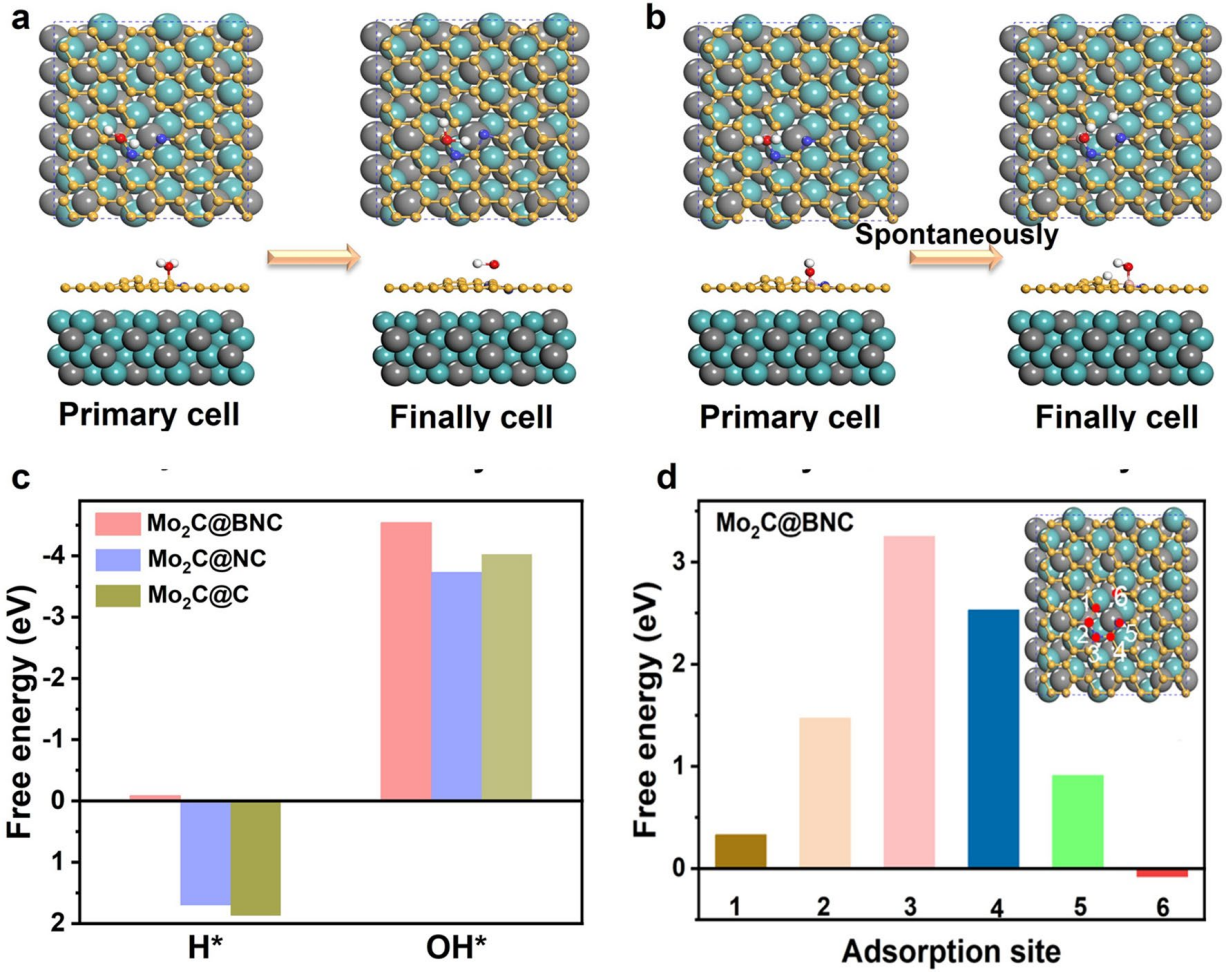
DFT calculation results of the HER process on the catalyst surfaces. H_2_O cleaving process on the surfaces of **a** Mo_2_C@NC and **b** Mo_2_C@BNC. **c** OH^*^ and H^*^ adsorption energies for the Mo_2_C@NC and Mo_2_C@BNC. **d** Calculated H^*^ free energy (Δ*G*_H_^*^) for different sites. Yellow: C atom, blue: N atom, pink: B atom, cyan: Mo atom. (Color figure online)

DFT calculations were performed to gain atomic insights into the synergistic effect of B doping on the HER activity. Figure 5a–b revealed that H_2_O is unfavorable for the adsorption on the Mo_2_C@NC substrate. However, the introduced B endows the superior ability of H_2_O adsorption and the following spontaneous decomposition on the Mo_2_C@BNC. This is confirmed by the hydrophilicity test of the samples. Mo_2_C@BNC possessed the best hydrophilicity with a water contact angle of 20.4°, which was smaller than that of Mo_2_C@NC (25.41°, Fig. S7), confirming the Mo_2_C@BNC exhibits better hydrophilicity than the Mo_2_C@NC. During this process, the reaction intermediates H^*^ and OH^*^ are prone to be adsorbed on the defective C site and B site, respectively. To identify the detailed roles of the dopants in the reaction, we calculated the possible adsorption of H^*^ and OH^*^ species on the theoretical models. As shown in Fig. 5c, the free energy for H^*^ adsorption (ΔG_H_^*^) on Mo_2_C@C surface is evaluated to be 1.87 eV, which is quite unfavorable for the hydrogen evolution reaction (Fig. S8a). The ΔG_H*_ on Mo_2_C@NC surface is 1.7 eV, implying that the N dopants could adjusted the electron distribution of external carbon layer, thus slightly improving the free energy of H^*^ adsorption (Fig. S9a). In contrast, the Mo_2_C@BNC displays a Pt-like H^*^ adsorption energy of − 0.085 eV (the C site) after B doping, which is an optimal state close to a thermo-neutral value. Furthermore, H^*^ free energies on different adsorption sites for the Mo_2_C@BNC surface were calculated to identify the active site in the reaction (the insert in Fig. 5d). The chosen theoretical model for the Mo_2_C@BNC is shown in the inset of Fig. 5d, in which the C site adjacent to B (Fig. S9b) is the most effective site for H^*^ adsorption (Fig. 5d). The significantly improved adsorption ability is attributed to the Co-introduction of B and N. When the electrons transfer from Mo to the carbon layer, due to the different electronegativity (B < C < N), the electrons will be redistributed between those non-metallic atoms, which can effectively activate the C atoms, making it a near-zero H^*^ adsorption Gibbs free energy.

Furthermore, the doping of B also strengthens the adsorption energy of OH^*^ (Fig. 5c). The best adsorption site for OH^*^ of the Mo_2_C@C and Mo_2_C@NC sample is the C site that near the H^*^ adsorption site (Figs. S8b and S9c). After B doping, the electron-deficient B atoms adsorbs water by coordinating with the lone pair of electrons, thus weakening the O−H bond to accelerate water dissociation. The best reaction center on the Mo_2_C@BNC surface becomes the B site (Fig. S9d). This is evidenced by the distance between the O atom from H_2_O and the active sites. It is calculated that the distance between the O atom and the adsorbed C site (d_O-C_) is 1.71 Å for Mo_2_C@C and 2.186 Å for Mo_2_C@NC. The value changes to 1.457 Å for the distance between the O atom and the adsorbed B site (d_O-B_) for Mo_2_C@BNC. As a result, the adsorption energy of the OH^*^ increases from − 4.0 and − 3.74 eV over the primary C site to − 4.54 eV over the B site. The enhanced adsorption energy of H^*^ and OH^*^ after the introduction of B is more favorable for H_2_O decomposition, which is clarified from the changes of density of projection state (DOS) of the Mo_2_C@BNC and Mo_2_C@NC samples (Fig. S10). Compared with the B-free sample, the H^*^-C and OH^*^-B interactions are strengthened in the B-doping sample. In short, DFT calculations confirm that the B doping greatly enhances the Volmer reaction in the HER process, making water cleaves into H^*^ and OH^*^. At the same time, the inert C atom is activated together with the B and N atoms and facilitated the Heyrovsky step. The synergistic effect between these non-metal B site and C site over the carbon shell thus greatly improving the HER activity.

## Conclusions

In summary, we have designed a Mo_2_C@BNC catalyst that N, B dual-doped ultrathin carbon shell encapsulated on Mo_2_C nanocrystals. The doping of N and B elements coupled with defects on the surface of the carbon layer, generates multiple active centers, which significantly enhances HER activity compared to the pristine samples. This catalyst delivers a current density of 10 mA cm^−1^ with a very low over-potential of 99 mV. More importantly, the catalytic activity even exceeds that of the commercial Pt/C catalyst at large current density, addressing one of the best non-noble metal based HER catalyst in alkaline solutions. Theoretical calculations reveal that the introduction of B can trigger the spontaneous decomposition of H_2_O and facilitate the Volmer reaction, simultaneously activating the adjacent C atom, resulting in a near-zero regulation of ΔG_H_^*^. This paper demonstrates the importance of multiple active centers concerning the alkaline HER activity and provides fresh insight to explore the acceleration of water dissociation via the effect of boron.

### Supplementary Information

Below is the link to the electronic supplementary material.

Supplementary file1 (PDF 1669 kb)

## References

[1] Li C, Clament Sagaya Selvam N, Fang J (2023). Shape-controlled synthesis of platinum-based nanocrystals and their electrocatalytic applications in fuel cells. Nano-Micro Lett..

[2] Jiang B, Tian D, Qiu Y, Song X, Zhang Y (2022). High-index faceted nanocrystals as highly efficient bifunctional electrocatalysts for high-performance lithium–sulfur batteries. Nano-Micro Lett..

[3] Yang Y, Yu Y, Li J, Chen Q, Du Y (2021). Engineering ruthenium-based electrocatalysts for effective hydrogen evolution reaction. Nano-Micro Lett..

[4] Li R, Xu H, Yang P, Wang D, Li Y (2021). Synergistic interfacial and doping engineering of heterostructured NiCo(OH)_x_-Co_y_W as an efficient alkaline hydrogen evolution electrocatalyst. Nano-Micro Lett..

[5] Guo B, Ding Y, Huo H, Wen X, Ren X (2023). Recent advances of transition metal basic salts for electrocatalytic oxygen evolution reaction and overall water electrolysis. Nano-Micro Lett..

[6] Chen Z, Duan X, Wei W, Wang S, Ni BJ (2020). Iridium-based nanomaterials for electrochemical water splitting. Nano Energy.

[7] Yin Q, Hill CL (2017). Water splitting: passing the acid test. Nat. Chem..

[8] Lu F, Zhou M, Zhou Y, Zeng X (2017). First-row transition metal based catalysts for the oxygen evolution reaction under alkaline conditions: basic principles and recent advances. Small.

[9] Tiwari JN, Sultan S, Myung CW, Yoon T, Li N (2018). Multicomponent electrocatalyst with ultralow Pt loading and high hydrogen evolution activity. Nat. Energy.

[10] Xie C, Chen W, Du S, Yan D, Zhang Y (2020). In-situ phase transition of WO_3_ boosting electron and hydrogen transfer for enhancing hydrogen evolution on Pt. Nano Energy.

[11] Chen J, Ha Y, Wang R, Liu Y, Xu H (2022). Inner Co synergizing outer ru supported on carbon nanotubes for efficient pH-universal hydrogen evolution catalysis. Nano-Micro Lett..

[12] Cheng Q, Hu C, Wang G, Zou Z, Yang H (2020). Carbon-defect-driven electroless deposition of Pt atomic clusters for highly efficient hydrogen evolution. J. Am. Chem. Soc..

[13] Zhang J, Wang E, Cui S, Yang S, Zou X (2022). Single-Atom Pt anchored on oxygen vacancy of monolayer Ti_3_C_2_T_x_ for superior hydrogen evolution. Nano Lett..

[14] Zhou M, Li H, Long A, Zhou B, Lu F (2021). Modulating 3d orbitals of Ni atoms on Ni-Pt edge sites enables highly-efficient alkaline hydrogen evolution. Adv. Energy Mater..

[15] Šljukić B, Vujković M, Amaral L, Santos DMF, Rocha RP (2015). Carbon-supported Mo_2_C electrocatalysts for hydrogen evolution reaction. J. Mater. Chem. A.

[16] Qiu Y, Wen Z, Jiang C, Wu X, Si R (2019). Rational design of atomic layers of Pt anchored on Mo_2_C nanorods for efficient hydrogen evolution over a wide pH range. Small.

[17] Fu W, Wang Y, Tian W, Zhang H, Li J (2020). Non-metal single-phosphorus-atom catalysis of hydrogen evolution. Angew. Chem. Int. Ed..

[18] Vrubel H, Hu X (2012). Molybdenum boride and carbide catalyze hydrogen evolution in both acidic and basic solutions. Angew. Chem. Int. Ed..

[19] Yang X, Cheng J, Yang X, Xu Y, Sun W (2023). Facet-tunable coral-like Mo_2_C catalyst for electrocatalytic hydrogen evolution reaction. Chem. Eng. J..

[20] Shi Y, Zhang B (2016). Recent advances in transition metal phosphide nanomaterials: synthesis and applications in hydrogen evolution reaction. Chem. Soc. Rev..

[21] Greeley J, Jaramillo TF, Bonde J, Chorkendorff IB, Norskov JK (2006). Computational high-throughput screening of electrocatalytic materials for hydrogen evolution. Nat. Mater..

[22] Yu F, Gao Y, Lang Z, Ma Y, Yin L (2018). Electrocatalytic performance of ultrasmall Mo_2_C affected by different transition metal dopants in hydrogen evolution reaction. Nanoscale.

[23] Wan C, Leonard BM (2015). Iron-doped molybdenum carbide catalyst with high activity and stability for the hydrogen evolution reaction. Chem. Mater..

[24] Wei H, Wang J, Lin Q, Zou Y, Chen X (2021). Incorporating ultra-small N-doped Mo_2_C nanoparticles onto 3D N-doped flower-like carbon nanospheres for robust electrocatalytic hydrogen evolution. Nano Energy.

[25] Lu Y, Yue C, Li Y, Bao W, Guo X (2021). Atomically dispersed Ni on Mo_2_C embedded in N, P co-doped carbon derived from polyoxometalate supramolecule for high-efficiency hydrogen evolution electrocatalysis. Appl. Catal. B Environ..

[26] Wang D, Liu T, Wang J, Wu Z (2018). N, P(S) Co-doped Mo_2_C/C hybrid electrocatalysts for improved hydrogen generation. Carbon.

[27] Liu Y, Yu G, Li GD, Sun Y, Asefa T (2015). Coupling Mo_2_C with nitrogen-rich nanocarbon leads to efficient hydrogen-evolution electrocatalytic sites. Angew. Chem. Int. Ed..

[28] Yang TT, Saidi WA (2020). Graphene activation explains the enhanced hydrogen evolution on graphene-coated molybdenum carbide electrocatalysts. J. Phys. Chem. Lett..

[29] Chen G, Wang T, Zhang J, Liu P, Sun H (2018). Accelerated hydrogen evolution kinetics on NiFe-layered double hydroxide electrocatalysts by tailoring water dissociation active sites. Adv. Mater..

[30] Ye S, Luo F, Xu T, Zhang P, Shi H (2020). Boosting the alkaline hydrogen evolution of Ru nanoclusters anchored on B/N–doped graphene by accelerating water dissociation. Nano Energy.

[31] Baek DS, Lee J, Kim J, Joo SH (2022). Metastable phase-controlled synthesis of mesoporous molybdenum carbides for efficient alkaline hydrogen evolution. ACS Catal..

[32] Lin Y, Zhou M, Tai X, Li H, Han X (2021). Analytical transmission electron microscopy for emerging advanced materials. Matter.

[33] Jiang H, Yan L, Zhang S, Zhao Y, Yang X (2021). Electrochemical surface restructuring of phosphorus-doped carbon@ MoP electrocatalysts for hydrogen evolution. Nano-Micro Lett..

[34] Huang Y, Gong Q, Song X, Feng K, Nie K (2016). Mo_2_C nanoparticles dispersed on hierarchical carbon microflowers for efficient electrocatalytic hydrogen evolution. ACS Nano.

[35] Qian G, Chen J, Yu T, Liu J, Luo L (2022). Three-phase heterojunction NiMo-based nano-needle for water splitting at industrial alkaline condition. Nano-Micro Lett..

[36] Zhang T, Wen G, Huang X, Zhong B, Yu H (2010). Preparation of high purity BCN hollow spheres by pyrolyzing a simple polymeric precursor. CrystEngComm.

[37] Yu X, Han P, Wei Z, Huang L, Gu Z (2018). Boron-doped graphene for electrocatalytic N_2_ reduction. Joule.

[38] Dogra A, Barlocco I, Singh A, Somodi F, Villa A (2020). Metal free alkene hydrogenation by B-doped graphitic carbon nitride. Catal. Sci. Technol..

[39] Ma R, Zhou Y, Chen Y, Li P, Liu Q (2015). Ultrafine molybdenum carbide nanoparticles composited with carbon as a highly active hydrogen-evolution electrocatalyst. Angew. Chem. Int. Ed..

[40] Li H, Yu F, Ling X, Wan H, Zhang M (2021). Dual-cation-doped MoS_2_ nanosheets accelerating tandem alkaline hydrogen evolution reaction. Nanotechnology.

[41] Li JS, Wang Y, Liu CH, Li SL, Wang YG (2015). Coupled molybdenum carbide and reduced graphene oxide electrocatalysts for efficient hydrogen evolution. Nat. Commun..

[42] Tabassum H, Zou R, Mahmood A, Liang Z, Guo S (2016). A catalyst-free synthesis of B, N co-doped graphene nanostructures with tunable dimensions as highly efficient metal free dual electrocatalysts. J. Mater. Chem. A.

[43] Zhang H, Ma Z, Duan J, Liu H, Liu G (2016). Active sites implanted carbon cages in core-shell architecture: highly active and durable electrocatalyst for hydrogen evolution reaction. ACS Nano.

[44] Xu H, Jia H, Fei B, Ha Y, Li H (2020). Charge transfer engineering via multiple heteroatom doping in dual carbon-coupled cobalt phosphides for highly efficient overall water splitting. Appl. Catal. B Environ..

[45] Wang X, Zheng Y, Sheng W, Xu ZJ, Jaroniec M (2020). Strategies for design of electrocatalysts for hydrogen evolution under alkaline conditions. Mater. Today.

[46] Shinagawa T, Garcia-Esparza A, Takanabe K (2015). Insight on Tafel slopes from a microkinetic analysis of aqueous electrocatalysis for energy conversion. Sci. Rep..

[47] Anjum MAR, Lee MH, Lee JS (2018). Boron- and nitrogen-codoped molybdenum carbide nanoparticles imbedded in a BCN network as a bifunctional electrocatalyst for hydrogen and oxygen evolution reactions. ACS Catal..

