# Supplementary material for: Synergistic Effect of Dual-Doped Carbon on Mo2C Nanocrystals Facilitates Alkaline Hydrogen Evolution
Source: Nanomicro Lett. 2023 Jul 3;15:166. doi: 10.1007/s40820-023-01135-0 (PMC10315362; doi:10.1007/s40820-023-01135-0)
Supplement: Supplementary file 1 — Supplementary file1 (PDF 1669 kb) [file 40820_2023_1135_MOESM1_ESM.pdf]

Supporting Information for

The Synergistic Effect of Dual-doped Carbon on Mo_2C Nanocrystals Facilitates Alkaline Hydrogen Evolution

Min Zhou [1, 3, *], Xiaoli Jiang [1, 2], Weijie Kong [3], Hangfei Li [3], Fei Lu [3], Xin Zhou [4], Yagang Zhang [1, 2, *]

[1] School of Materials and Energy, University of Electronic Science and Technology of China, Chengdu, 611731, P. R. China

[2] State Key Laboratory of Electronic Thin Films and Integrated Devices, University of Electronic Science and Technology of China, Chengdu, 610054, P. R. China

[3] College of Physical Science and Technology, Yangzhou University, Yangzhou, 225002, P. R. China

[4] Department of Chemical Engineering, Polytechnique Montréal, Montréal, Québec H3C 3A7, Canada

*Corresponding authors. E-mail: ygzhang@uestc.edu.cn (Y. Zhang); minzhou@yzu.edu.cn (M. Zhou)

Supplementary Figures and Tables

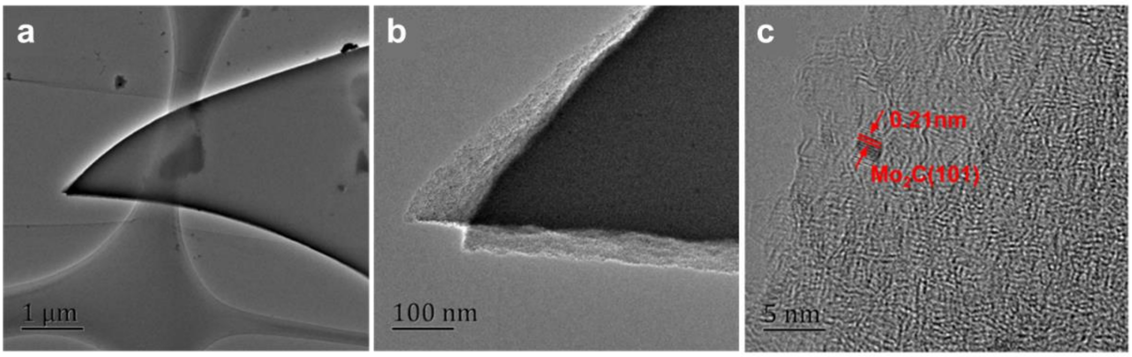

Fig. S1 Glucose as carbon source. (**a, b**) TEM images of Mo_2C@C, (**c**) HR-TEM image

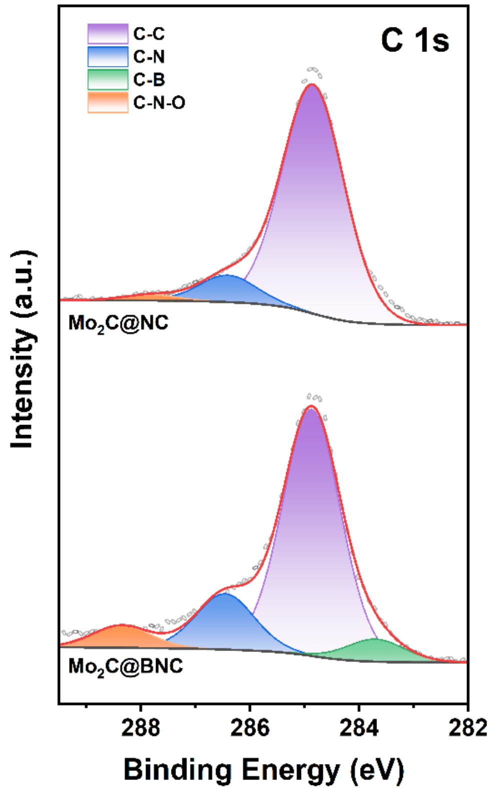

Fig. S2 XPS spectra of Mo_2C@NC and Mo_2C@BNC for C 1s

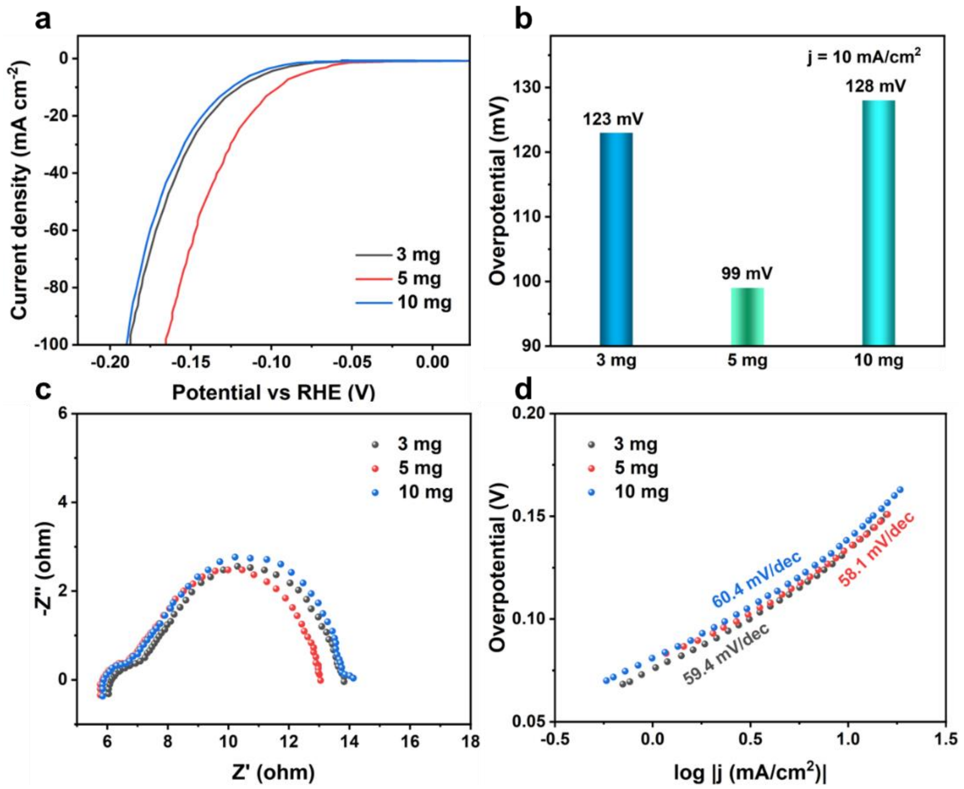

Fig. S3 (**a**) LSV curves of different contents of doped B. (**b**) Overpotential (η_{10}) comparison histogram. (**c**) Electrochemical impedance (EIS) plots. (**d**) Tafel slope plots

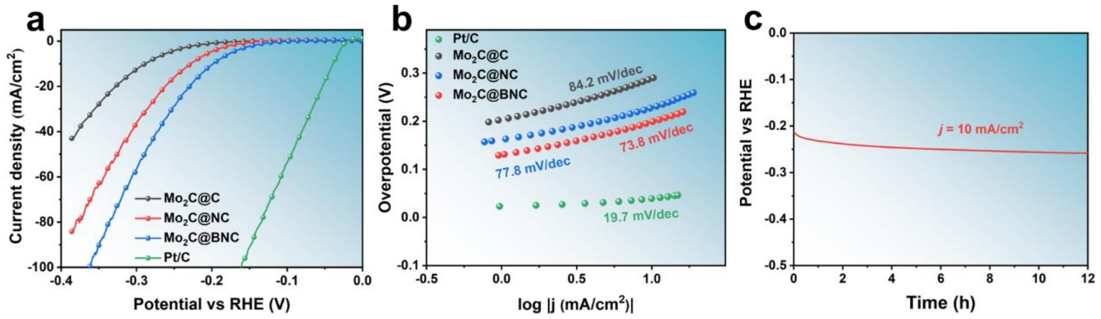

Fig. S4 (**a**) LSV curves of Mo_2C@C, Mo_2C@NC, Mo_2C@BNC and Pt/C catalysts in 0.5 M H_2SO_4 electrolyte. (**b**) Tafel slope curve. (**c**) V-t plot of constant potential for Mo_2C@BNC catalyst

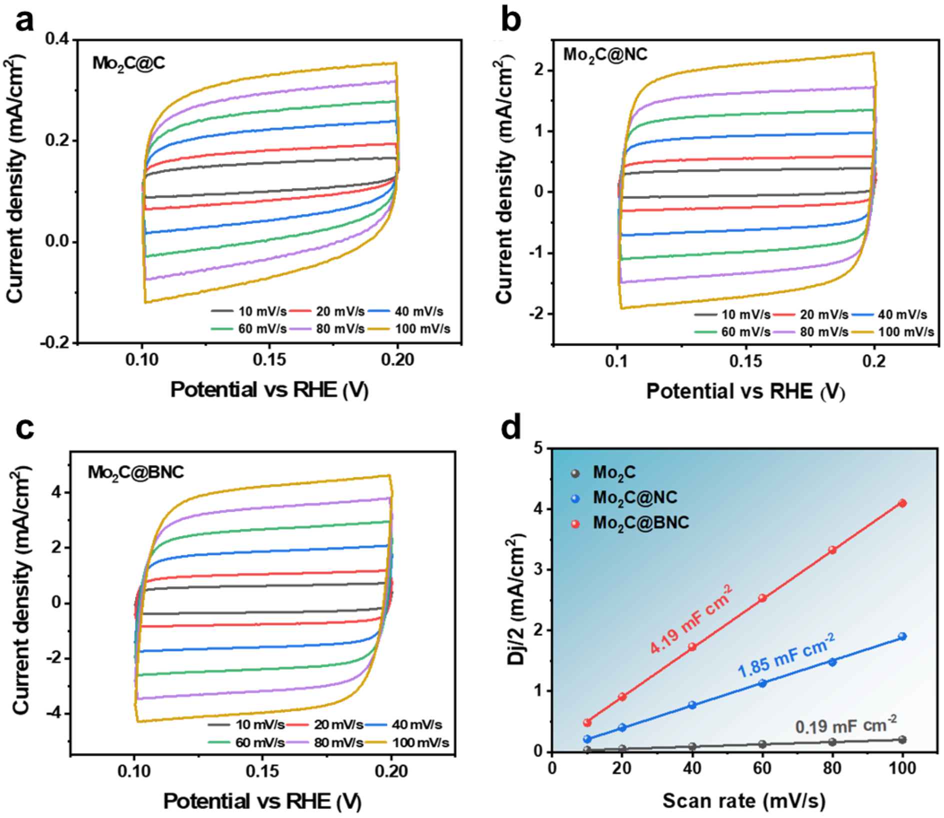

Fig. S5 CV curves of (**a**) Mo_2C@C, (**b**) Mo_2C@NC and (**c**) Mo_2C@BNC. (d) Current density versus scan rates in 1 M KOH solution

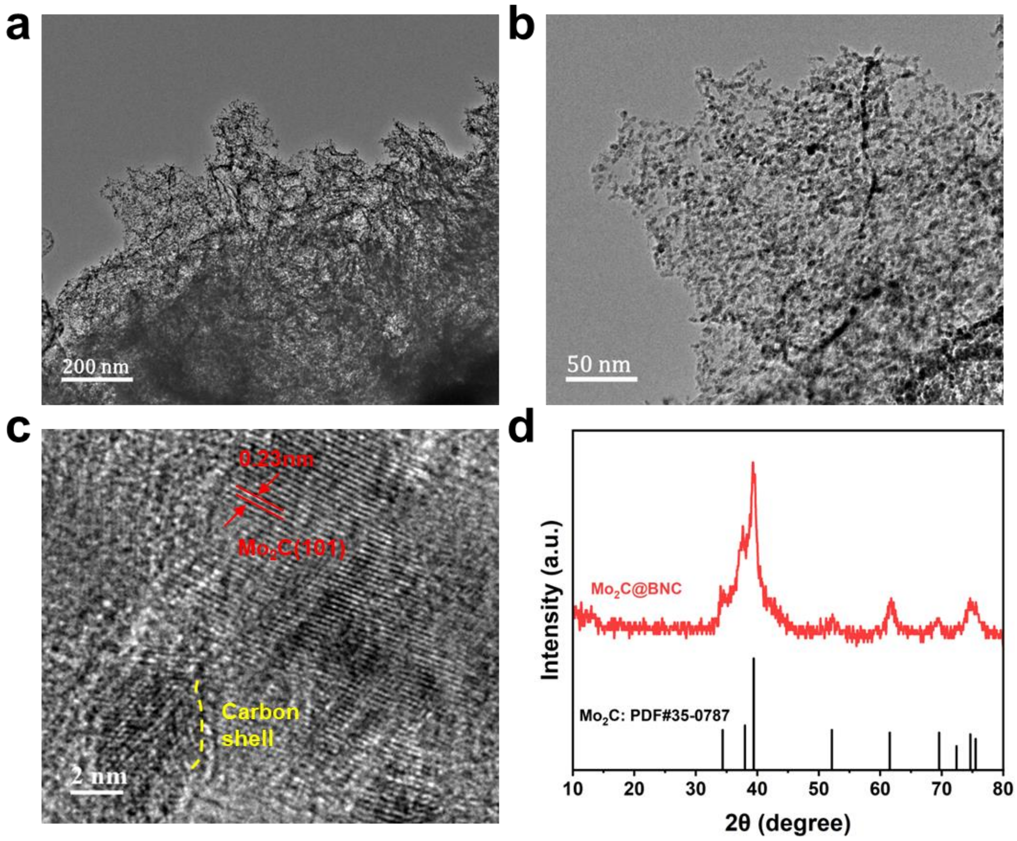

Fig. S6 The structural characterization of Mo_2C@BNC catalysts after durability test. (**a-b**) TEM images, (**c**) HRTEM image and (**d**) XRD pattern

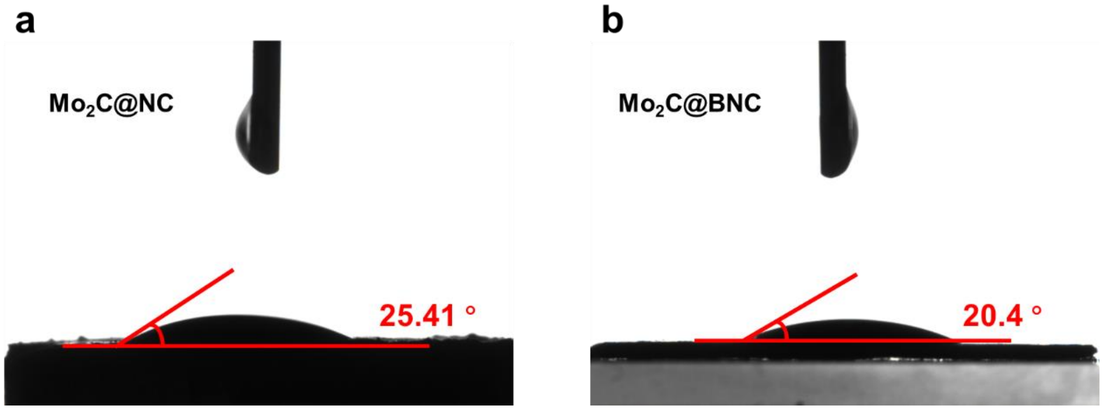

Fig. S7 The contact angles of (**a**) Mo_2C@BNC and (**b**) Mo_2C@NC

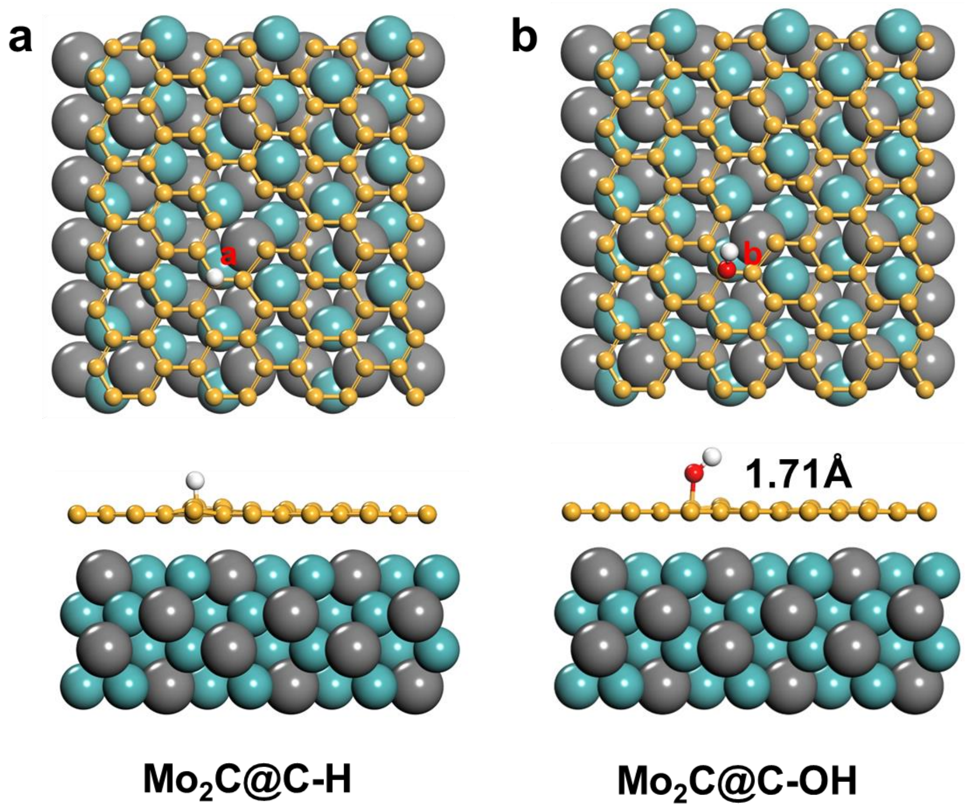

Fig. S8 (**a-b**) Optimized H^* and OH^* adsorption on Mo_2C@C

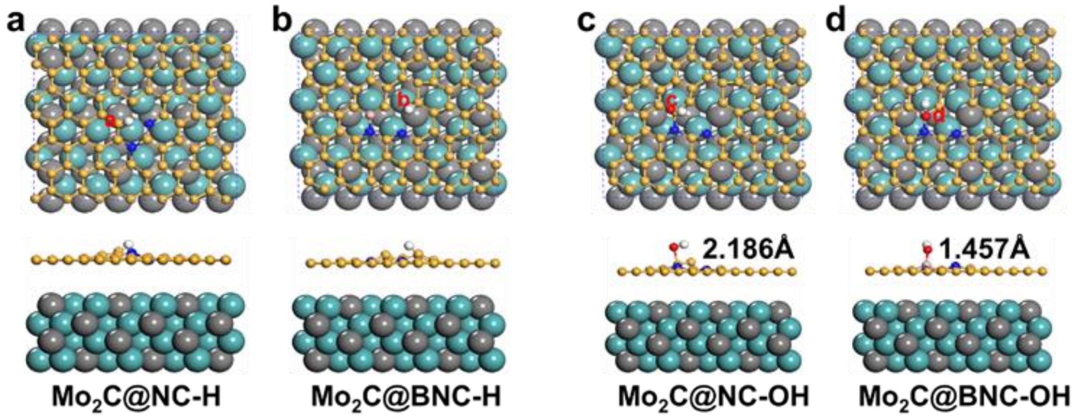

Fig. S9 (**a-d**) Optimized H^* and OH^* adsorption on Mo_2C@NC and Mo_2C@BNC

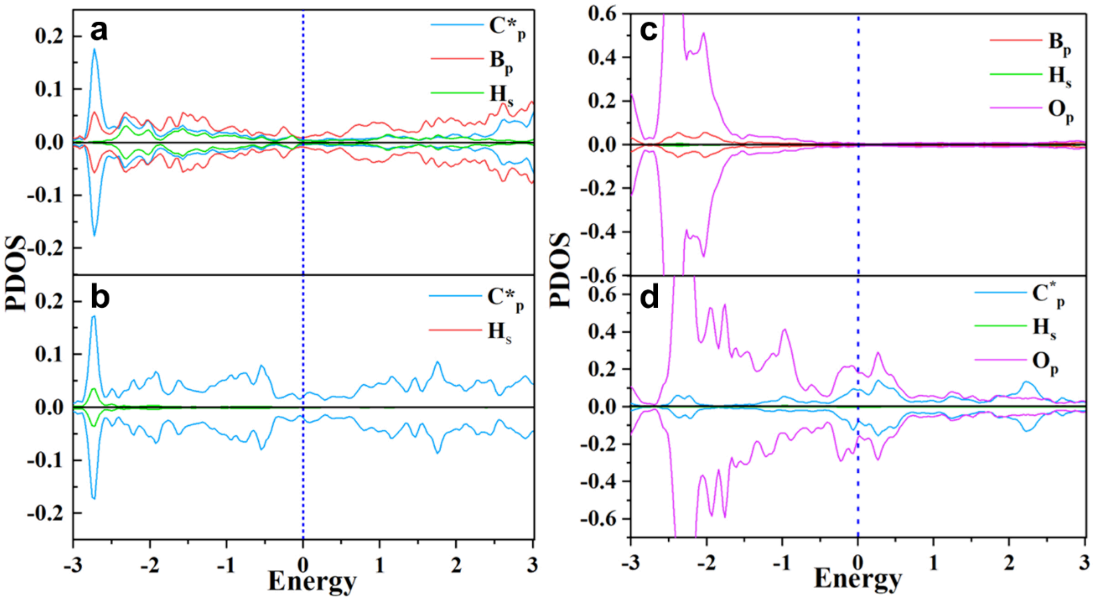

Fig. S10 Density of projection states (DOS) of samples. *: active sites. (**a**) the DOS of adsorbed H* for Mo_2C@BNC. (**b**) the DOS of adsorbed H* for Mo_2C@NC. (**c**) the DOS of adsorbed OH* for Mo_2C@BNC. (**d**) the DOS of adsorbed OH* for Mo_2C@NC

Table S1 Elemental Analysis Results of Mo_2C@BNC Catalysts

**sample**	**Mo (wt%)**	**C (wt%)**	**B (wt%)**	**N (wt%)**
Mo_2C@BNC	44.82	36.44	10.32	8.42

Determined by Elemental mapping analysis.

Table S2 Proportions of various N in Mo_2C@BNC and Mo_2C@NC samples

	**Mo2C@BNC**	**Mo_2C@NC**
Graphitic N	15.09	15.41
Pyrrolic N	35.99	60.09
Pyridinic N	38.97	24.50
N-B	9.95	0

Table S3 Comparisons of the HER performance of Mo_2C@BNC and other electrocatalysts

Catalyst	Electrolyte	Overpotential (@10 mA/cm²)	Overpotential (@50 mA/cm²)	Tafel slope (mV/dec)	Electrode	Loading mass (mg/cm²)	Refs.
Mo/Co@N-C	1 M KOH	157 mV	250 mV	148	GC	0.7	1
Co-Mo_2C-CN_x	1 M KOH	92 mV	175 mV	89	GC	0.781	2
Ni-Mo_2C	1 M KOH	172mV	~	68	GC	0.25	3
MMC-H_2	1 M KOH	96 mV	~	37	GC	0.78	4
Mo_2C	1 M KOH	116 mV	> 400 mV	65	NF	~	5
3D Mo_2C	1 M KOH	110 mV	199 mV	73.9	NF	0.26	6
MoC_x	1 M KOH	151 mV	218 mV	59	GC	0.8	7
Mo_2C/NCF	1 M KOH	100 mV	~	65	GC	0.28	8
$Co_{0.3}Mo_{1.69}C$/ Mxene/NC	1 M KOH	75 mV	~	43	GC	0.2	9
$Mo_1N_1C_2$	0.1 M KOH	132 mV	~	90	GC	0.408	10
Co/β-Mo_2C@N-CNTs	1 M KOH	170 mV	280 mV	92	GC	0.014	11
Ni/Mo_2C-PC	1 M KOH	179 mV	~	101	GC	0.25	12
Mo_2C@BNC	1 M KOH	99 mV	142 mV	58.1	GC	0.54	This work
Co_6W_6C	1 M KOH	50 mV	116 mV	40.99	GC	0.51	13
Pt–Ni_4Mo/CNT	1M KOH	18.6 mV	75 mV	37.4	GC	0.014	14
Ni@CNTs-Mo_xC/Ni_2P	1M KOH	54 mV	120 mV	54	NF	~	15
Fe/SAs@Mo-based-HNSs	1M KOH	38.5 mV	80 mV	35.6	CP	0.67	16

Supplementary References

[S1] C. Wu, D. Liu, H. Li, J. Li, Molybdenum Carbide-Decorated Metallic Cobalt@ Nitrogen-Doped Carbon Polyhedrons for Enhanced Electrocatalytic Hydrogen Evolution. Small **14**(16), e1704227 (2018). https://doi.org/10.1002/smll.201704227

[S2] P. Zhang, Y. Liu, T. Liang, E. H. Ang, X. Zhang et al., Nitrogen-doped carbon wrapped Co-Mo_2C dual Mott–Schottky nanosheets with large porosity for efficient water electrolysis. Appl. Catal. B Environ. **284**, 119738 (2021). https://doi.org/10.1016/j.apcatb.2020.119738

[S3] W. Wang, L. Yang, J. Chen, S. Wu, M. Chen et al., Realizing electronic modulation on Mo sites for efficient hydrogen evolution reaction. J. Mater. Chem. A **8**(35), 18180–18187 (2020). https://doi.org/10.1039/D0TA05957A

[S4] D. S. Baek, J. Lee, J. Kim, S. H. Joo, Metastable Phase-Controlled Synthesis of Mesoporous Molybdenum Carbides for Efficient Alkaline Hydrogen Evolution. ACS Catal. **12**(12), 7415–7426 (2022). https://doi.org/10.1021/acscatal.2c01772

[S5] S. Hussain, D. Vikraman, A. Feroze, W. Song, K. S. An et al., Synthesis of Mo2C and W2C Nanoparticle Electrocatalysts for the Efficient Hydrogen

Evolution Reaction in Alkali and Acid Electrolytes. Front. Chem. **7**, 716 (2019). https://doi.org/10.3389/fchem.2019.00716

[S6] X. Yang, J. Cheng, X. Yang, Y. Xu, W. Sun et al., Facet-tunable coral-like Mo_2C catalyst for electrocatalytic hydrogen evolution reaction. Chem. Eng. J. **451**(4), 138977 (2023). https://doi.org/10.1016/j.cej.2022.138977

[S7] H. B. Wu, B. Y. Xia, L. Yu, X. Y. Yu, X. W. Lou, Porous molybdenum carbide nano-octahedrons synthesized via confined carburization in metal-organic frameworks for efficient hydrogen production. Nat. Commun. **6**, 6512 (2015). https://doi.org/10.1038/ncomms7512

[S8] Y. Huang, Q. Gong, X. Song, K. Feng, K. Nie et al., Mo_2C Nanoparticles Dispersed on Hierarchical Carbon Microflowers for Efficient Electrocatalytic Hydrogen Evolution. ACS Nano **10**(12), 11337–11343 (2016). https://doi.org/10.1021/acsnano.6b0658

[S9] X. Wu, S. Zhou, Z. Wang, J. Liu, W. Pei et al., Engineering Multifunctional Collaborative Catalytic Interface Enabling Efficient Hydrogen Evolution in All pH Range and Seawater. Adv. Energy Mater. **9**(34), 1901333 (2019). https://doi.org/10.1002/aenm.201901333

[S10] W. Chen, J. Pei, C. T. He, J. Wan, H. Ren et al., Rational Design of Single Molybdenum Atoms Anchored on N-Doped Carbon for Effective Hydrogen Evolution Reaction. Angew. Chem. Int. Ed. **56**(50), 16086–16090 (2017). https://doi.org/10.1002/anie.201710599

[S11] T. Ouyang, Y. Q. Ye, C. Y. Wu, K. Xiao, Z. Q. Liu, Heterostructures Composed of N-Doped Carbon Nanotubes Encapsulating Cobalt and β-Mo_2C Nanoparticles as Bifunctional Electrodes for Water Splitting. Angew. Chem. Int. Ed. **58**(15), 4923–4928 (2019). https://doi.org/10.1002/anie.201814262

[S12] Z. Y. Yu, Y. Duan, M. R. Gao, C. C. Lang, Y. R. Zheng et al., A one-dimensional porous carbon-supported Ni/Mo_2C dual catalyst for efficient water splitting. Chem. Sci. **8**(2), 968–973 (2017). https://doi.org/10.1039/C6SC03356C

[S13] G. Meng, Y. Chen, R. Wang, L. Zhu, H. Yao et al., Co-W Bimetallic Carbide Nanocatalysts: Computational Exploration, Confined Disassembly–Assembly Synthesis and Alkaline/Seawater Hydrogen Evolution. Small **18**(48) 2204443 (2022). https://doi.org/10.1002/smll.202204443

[S14] J. Liu, Z. Wang, X. Wu, D. Zhang, Y. Zhang et al., Pt doping and strong metal–support interaction as a strategy for NiMo-based electrocatalysts to boost the hydrogen evolution reaction in alkaline solution. J. Mater. Chem. A **10**, 15395-15401 (2022). https://doi.org/10.1039/D2TA03934F

[S15] J. Wang, D. T. Tran, K. Chang, S. Prabhakaran, J. Zhao et al., Hierarchical Ni@CNTs-bridged Mo_xC/Ni_2P heterostructure micro-pillars for enhanced seawater splitting and Mg/seawater battery. Nano Energy **111**, 108440 (2023).

https://doi.org/10.1016/j.nanoen.2023.108440

[S16] F. Lyu, S. Zeng, Z. Jia et al., Two-dimensional mineral hydrogel-derived single atoms-anchored heterostructures for ultrastable hydrogen evolution. Nat Commun **13**, 6249 (2022). https://doi.org/10.1038/s41467-022-33725-8
